# ErbB1-4-dependent EGF/neuregulin signals and their cross talk in the central nervous system: pathological implications in schizophrenia and Parkinson's disease

**DOI:** 10.3389/fncel.2013.00004

**Published:** 2013-02-13

**Authors:** Yuriko Iwakura, Hiroyuki Nawa

**Affiliations:** Division of Molecular Neurobiology, Brain Research Institute, Niigata UniversityNiigata, Japan

**Keywords:** ErbB1-4, dopamine, GABA, virokine, schizophrenia, Parkinson's disease

## Abstract

Ligands for ErbB1-4 receptor tyrosine kinases, such as epidermal growth factor (EGF) and neuregulins, regulate brain development and function. Thus, abnormalities in their signaling are implicated in the etiology or pathology of schizophrenia and Parkinson's disease. Among the ErbB receptors, ErbB1, and ErbB4 are expressed in dopamine and GABA neurons, while ErbB1, 2, and/or 3 are mainly present in oligodendrocytes, astrocytes, and their precursors. Thus, deficits in ErbB signaling might contribute to the neurological and psychiatric diseases stemming from these cell types. By incorporating the latest cancer molecular biology as well as our recent progress, we discuss signal cross talk between the ErbB1-4 subunits and their neurobiological functions in each cell type. The potential contribution of virus-derived cytokines (virokines) that mimic EGF and neuregulin-1 in brain diseases are also discussed.

## Introduction

**Figure 1:**
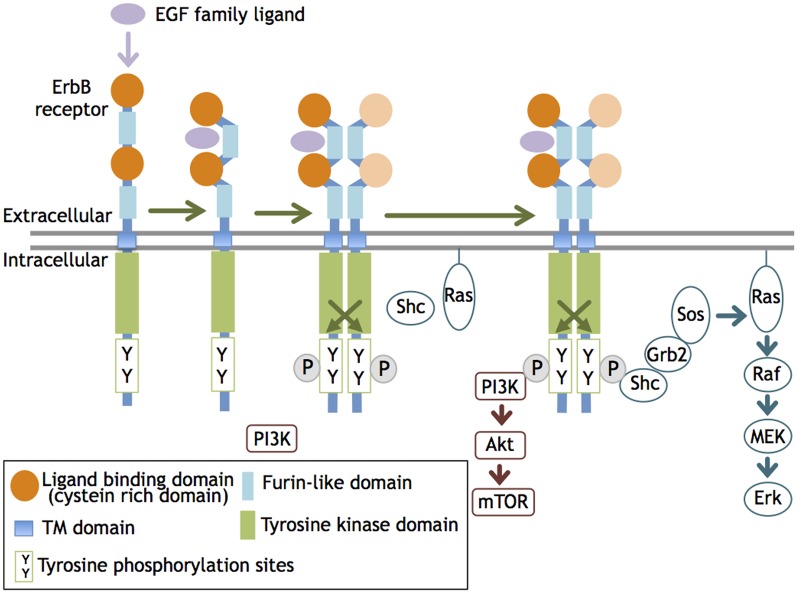
**ErbB receptor dimerization and activation.** The ligand interaction with ErbB 1, 3, and 4 increases their affinity and induces homo- or heterodimerization of ErbB1-4. This dimerization activates the tyrosine kinase domain and allows it to phosphorylate the cytoplasmic region of the ErbB partner. The phosphorylated tyrosine residues recruit various adaptors/effectors that induce intracellular signals.

ErbB molecules are membrane-spanning receptor tyrosine kinases that act on epidermal growth factor (EGF) and its derivatives. The ErbB family consists of four members, ErbB1, B2, B3, and B4, that were originally identified in vertebrates (mammals) and share significant structural homology with members of the ErbB family (Downward et al., 1984; Schechter et al., 1984; Semba et al., 1985; Kraus et al., 1989; Plowman et al., 1993). ErbB1 homolog is identified also in invertebrates, *C. elegans* (LET-23; Aroian et al., 1990) and Drosophila (DER; Schejter and Shilo, 1989). ErbB receptor family members are distributed in many organs and cell types originating from ectodermal and mesodermal tissues and have functions in various cellular processes/functions such as proliferation, growth, migration, and adhesion. Upon binding to its ligand such as EGF and neuregulin-1 (NRG1), the ErbB receptor undergoes tertiary structural alterations in the juxtamembrane region and increases its affinity for another an ErbB molecule, leading to homo- or heterodimerization (Olayioye et al., 2000). This dimerization allows the kinase domain to phosphorylate the ErbB partner. The phosphorylated tyrosine residues recruit adaptor/effecter molecules, such as phosphatidylinositol 3-kinase (PI3K) subunit p85, Src, and Shc, and transmit signals to these transducers. As overexpression of ErbB receptors results in ligand-independent dimerization and auto-phosphorylation, receptor dimerization, rather than the activation of the kinase domain, is thought to limit ErbB signaling (i.e., phosphorylation) (Figure 1).

**Figure 2:**
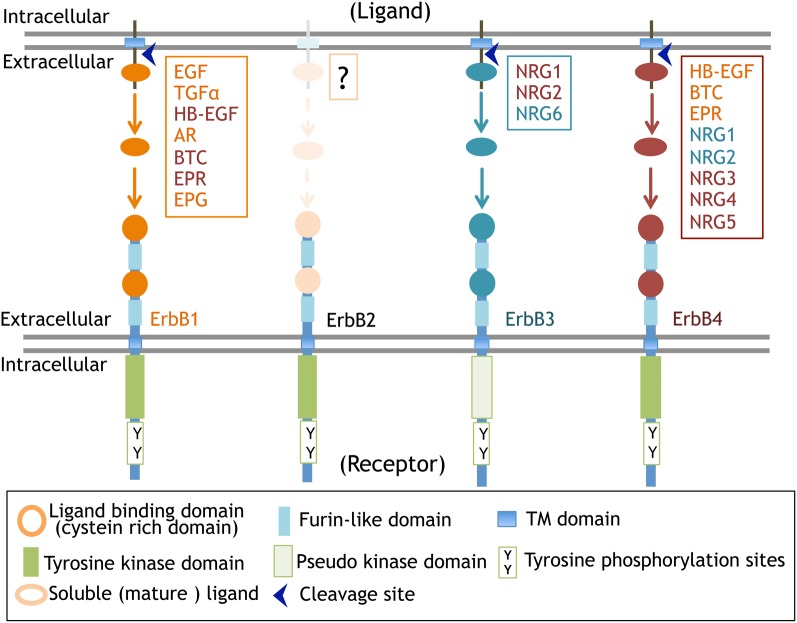
**ErbB receptors and their ligands.** The molecular structure of ErbB receptors and proteolytic processing of their ligands are displayed.

The primary structure of ErbB1 (EGFR, HER1) was first elucidated among ErbBs. The oncogene *v-erbB* was identified in avian erythroblastic leukemia virus. The ortholog and proto-oncogene of *c-erbB* was determined to be the gene for EGF receptor, *erbB1* (Downward et al., 1984). Following this discovery, homologous gene cloning led to the identification of the other of ErbB family members, including ErbB2 (HER2, Neu), ErbB3 (HER3), and ErbB4 (HER4) (Schechter et al., 1984; Semba et al., 1985; Kraus et al., 1989; Plowman et al., 1993). This family shares 40–50% structural homology in the extracellular domains and 60–80% in the intracellular domains (Figure 2, Table 1).

**Table 1:** **ErbB molecules and their ligands and adaptors/effectors**.

**ErbB receptor**	**Nomenclature**	**Binding partner**	**Ligand**		**Major signaling molecules**	**Other binding proteins**
ErbB1	EGF receptor (EGFR), HER1	ErbB1	EGF	EPG	PI3K-AKT	Cbl
		ErbB2	TGFα	EPR	Ras-MAPK	STAT3
		ErbB3	HB-EGF		PLCγ-PKC	
		ErbB4	AR		Crk FAK JAK	
			BTC		Src PTEN	
ErbB2	Neu, HER2	ErbB1	Unknown		PI3K-AKT	Erbin
		ErbB3			Ras-MAPK	
		ErbB4			FAK Src	
ErbB3	HER3	ErbB1	NRG1		PI3K-AKT	Cbl
		ErbB2	NRG2		Ras-MAPK	EBP1
		ErbB4	NRG6		PLCγ-PKC	TENC1
					Crk ITK JAK Lyn Src VAV	
ErbB4	HER4	ErbB1	HB-EGF	NRG2	PI3K-AKT	N-Cor
		ErbB2	BTC	NRG3	Ras-MAPK	PSD95
		ErbB3	EPG	NRG4	JAK Src Ptprz	STAT5
		ErbB4	NRG1	NRG5		TAB2

## The molecular features of the ErbB family

Ligands for ErbB receptors can be classified into two groups: the EGF family and the NRG family (Falls, 2003; Higashiyama et al., 2008; Mei and Xiong, 2008). The EGF family consists of transforming growth factor alpha (TGFα; Derynck et al., 1984), heparin-binding EGF-like growth factor (HB-EGF; Higashiyama et al., 1991), amphiregulin (AR; Shoyab et al., 1989), epiregulin (EPR; Toyoda et al., 1995), betacellulin, (BTC; Sasada et al., 1993; Shing et al., 1993), and epigen (EPG; Strachan et al., 2001). The NRG family includes NRG1 (Brockes and Kintner, 1986; Holmes et al., 1992; Peles et al., 1992; Falls et al., 1993; Ho et al., 1995), NRG2 (NTAK; Higashiyama et al., 1997), NRG3, NRG4 (Hobbs et al., 2002), NRG5 (tomoregulin; Uchida et al., 1999), and NRG6 (neuroglycan C; Kinugasa et al., 2004). Most of these ligands are initially synthesized as large membrane-anchored precursors that are processed into secretable and soluble forms and then liberated into the extracellular space where they interact with ErbB receptors (Figure 2). In contrast to this process of “endocrine signaling,” these precursors are also thought to mediate “juxtacrine signaling” during cell–cell communication; the membrane-anchored precursors directly bind to ErbB receptors on the other side of the cell surface (Ono et al., 1994). Please read the details of “juxtacrine signaling” in other reviews (Iwamoto and Mekada, 2000; Singh and Harris, 2005).

Among the ErbB receptors, ErbB3 lacks the active kinase domain and is unable to phosphorylate ErbB in this dimer complex. However, upon ligand binding, ErbB3 associates with the heterodimer complex containing the other ErbB and is phosphorylated by the partner ErbB kinase, leading to signal transduction by ErbB3 (Sierke et al., 1997). Conversely, ErbB2 harbors an active kinase domain, but its high-affinity ligands remain unknown (Cho et al., 2003; Garrett et al., 2003). The ErbB1 and ErbB2 genes are often amplified and overexpressed in various cancer cells, resulting in self-dimerization and auto-phosphorylation in a ligand-independent manner. For instance, ErbB2 is amplified in 3% of lung cancers, 30% of breast cancers, 20% of gastric cancers, and 60% of ovarian cancers. The combination of the ErbB1-4 subunits during heterodimerization does not appear to be limited; NRG1-bound ErbB4 can associate with ErbB1 to form a heterodimer, even though ErbB1 is not activated by EGF (Liu et al., 2012). NRG1 mimics EGF signaling through ErbB1 phosphorylation in ErbB4:ErbB1 heterodimer complexes. In the ErbB4:ErbB1 heterodimer, NRG promotes more threonine phosphorylation and less tyrosine phosphorylation of ErbB1, which results in Shc/Grb2 recruitment, than does EGF (Olayioye et al., 1998). Therefore, even within the context of the same heterodimer, distinct ligands can differentially impact receptor signaling (Moghal and Sternberg, 1999). Of note, external stimuli can also affect partnership during ErbB heterodimerization. Glucocorticoids can interfere with organized ErbB receptor dimerization in lung cells, leading to a switch from ErbB1:ErbB4 to ErbB2:ErbB4 heterodimer expression (Table 1; Dammann et al., 2006). In addition to the signaling complexity and diversity of ErbB heterodimerization, the individual *erbB* genes or their products are subjected to alternative splicing or proteolytic processing, resulting in truncated isoforms lacking the kinase domain or ligand-binding domain. These truncated isoforms function as an enhancer of tumorigenesis, a receptor decoy or a transcription factor (see below; Yamazaki et al., 1988; Lee et al., 2001; Vidal et al., 2005; Sundvall et al., 2007; Lin et al., 2008; Xia et al., 2011; Ward et al., 2012).

## Cell signaling and functions of individual ErbB molecules

All ErbB molecules are expressed not only in peripheral tissues but also in various neural cells (Table 2). In the view of their functionality in the brain, we need to consider which ErbB subtype is expressed, where it is expressed, and with which ErbB molecule it colocalizes or dimerizes. As discussed above, the phosphorylated ErbB partner determines the functional nature of signaling, irrespective of the ErbB ligand. In this context, it is not true that NRG only evokes the signals of its receptor, ErbB3 and/or ErbB4.

**Table 2:** **Brain distribution and functions of ErbB1-4**.

**ErbB receptor**	**Tissue**	**Cell type**	**Function**
ErbB1	Subventrucular zone	Neural stem cell	Proliferation/migration
	Midbrain	Dopaminergic neuron	Survival/development
	Cortex, hippocampus	GABAergic neuron Astrocyte	Regulation of synaptic function Proliferation/differentiation
	Cerebellum	Purkinje cell Granule cell Astrocyte	Development/proliferation
	Pituitary grand	Lactotroph	Production/release of cortisol and prolactin
ErbB2	Cerebellum, cortex, hippocampus, midbrain, etc.	Oligodendrocyte Astrocyte Radial glia	Proliferation/differentiation
ErbB3	Cortex, hippocampus, etc.	Oligodendrocyte	Maturation/myelination
ErbB4	Cortex, hippocampus	GABAergic neuron Astrocyte Oligodendrocyte	Attenuates synaptic function Proliferation/differentiation
	Cerebellum	Granule cell	Regulation of synaptic function
	Midbrain	Dopaminergic neuron	Survival, attenuates synaptic function

## ErbB1 (EGFR, HER1)

**Figure 3:**
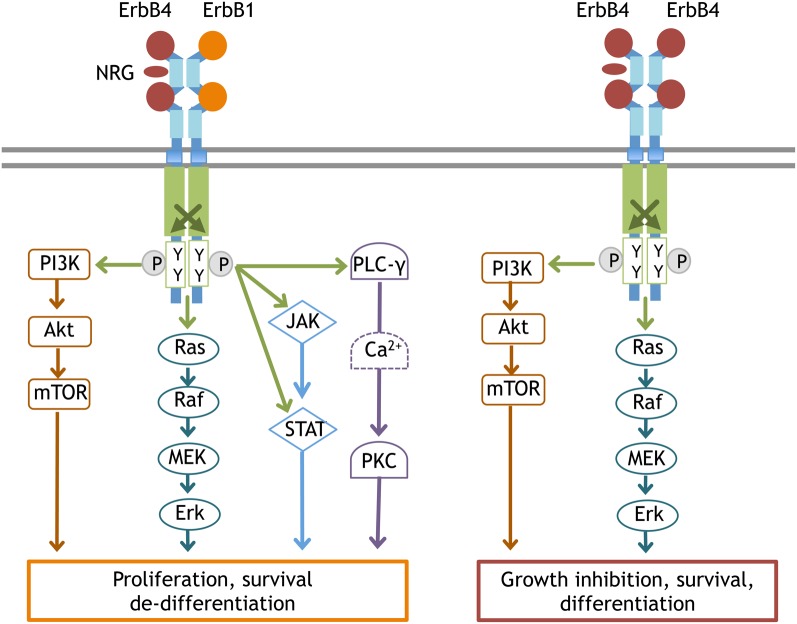
**Typical signal transduction from the ErbB4:ErbB1 and ErbB4:ErbB4 complex.** Once ErbB1 is phosphorylated by the partner ErbB, the following signal cascades are activated; **(1)** In the PLCγ-PKC pathway, phosphorylated ErbB1 recruits and associates with PLCγ. As a result, PLCγ itself is phosphorylated to activate DAG/IP3 signaling (Chen et al., 1996). **(2)** In the Ras-MAPK pathway, phosphorylated ErbB1 associates with Shc and interacts with Grb2/Sos1. Activated Sos1 triggers GDP/GTP exchange in Ras and activates Ras, driving the sequential kinase reactions of Raf(MAPKKK), MEK(MAPKK), and Erk(MAPK). **(3)** In the PI3K-Akt pathway, the activated ErbB dimer interacts with Grb2/Gab1 and forms complexes with activated PI3Kinase, leading to the conversion of PIP2 to PIP3 and Akt activation. **(4)** In the JAK-STAT pathway, ErbB kinase phosphorylates and induces JAK to bind to ErbB1. Activated JAK phosphorylates STAT and allows STAT to homodimerize and translocate into the nucleus. Once ErbB4 is phosphorylated by ErbB4, the signaling cascades linked to differentiation become activated, notably the PI3K-Akt pathway and the Ras-MAPK pathway with longer durations.

ErbB1 signaling links to a large variety of cellular functions, such as cell survival and proliferation. The down-stream signals linked to ErbB include the phospholipase Cγ (PLCγ)- protein kinase C (PKC), Ras- mitogen-activated protein kinase (MAPK), PI3K-Akt, and janus kinase 2 (JAK2)-STAT3 pathways (Figure 3). The Ras-MAPK pathway is implicated in cell proliferation and differentiation, while the PI3K-Akt pathway is involved in cell growth and anti-apoptotic processes and the PLCγ-PKC pathway contributes to cell migration and division. The subcellular localization and protein levels of these adaptors/effectors appear to determine the distinct features of ErbB1 down-stream signals. ErbB1 has a deletion mutant, EGFRvIII (also known as ΔEGFR, type3 EGFR, de 2–7 EGFR, EGFR^*^), which lacks extracellular domain of EGFR (Yamazaki et al., 1988). In addition, alternative splicing and protein processing produce soluble EGFR isoform (sEGFR). sEGFR lacks intracellular domain (Flickinger et al., 1992; Rose-John and Heinrich, 1994; Perez-Torres et al., 2008). These truncated ErbB1 contribute to tumorigenesis, but their role in brain is not fully understood (Baron et al., 2003; Gan et al., 2009).

In the central nervous system, ErbB1 protein levels are the highest during the gestational stages and gradually decline during development. Consistent with this expression pattern, neural stem cells in the subventricular zone (SVZ) are highly enriched with ErbB1 (Abe et al., 2009a). ErbB1 activation triggers the proliferation and migration of neural stem cells and their immediate decedents (Aguirre et al., 2005, 2010). In addition to these undifferentiated neural cells, several types of differentiated neurons also maintain expression at postnatal stages. In the midbrain region, the nigra-striatal dopamine neurons express ErbB1 together with ErbB4 (Abe et al., 2009a). ErbB1 activation contributes to the survival and postnatal development of dopaminergic neurons, although the molecular nature of the endogenous ErbB1 ligands has not been fully identified (Iwakura et al., 2005, 2011; Namba et al., 2009). Various types of GABAergic neurons also carry ErbB1 receptors. Interneurons in the hippocampus and neocortex as well as cerebella Purkinje cells express ErbB1 (Werner et al., 1988; Seroogy et al., 1995; Namba et al., 2006; Nagano et al., 2007; Abe et al., 2009b). In contrast to its action on dopaminergic neurons, the activation of ErbB1 in GABAergic neurons induces their de-differentiation, as seen in peripheral cancer cells (Namba et al., 2006; Nagano et al., 2007). The differences in the biological activities of EGF/ErbB1 signaling between these two neuronal populations are presumably attributed to differences in the ErbB partner. The signal pathways of ErbB1:ErbB1 homodimers and ErbB1:ErbB4 heterodimers differ significantly as discussed above. In addition to these neurons, astrocytes and their precursors express ErbB1, which is markedly enhanced in response to injury-associated astrogliosis (Liu et al., 2006). ErbB1 is also expressed in the pituitary and regulates the production and release of cortisol and prolactin (Cooper et al., 2011; Dahlhoff et al., 2011). ErbB1 in the hypothalamus reacts with TGFα, which is produced in the suprachiasmatic nucleus, and regulates circadian rhythm (Kramer et al., 2001; Snodgrass-Belt et al., 2005).

## ErbB2 (HER2, Neu)

ErbB2 signals mainly link to the Ras-MAPK pathway and the PI3K-Akt pathway, leading to cell proliferation. Therefore, at the postnatal stages, ErbB2 levels are limited to postmitotic neurons or glial cells (Abe et al., 2009a). There are a variety of truncated ErbB2 isoforms that are produced by alternative splicing and metalloprotease digestion (Cappuzzo et al., 2012; Tse et al., 2012; Ward et al., 2012). p110ErbB2 (611CTF) and p95ErbB2 (CTF648) both lack the extracellular domain and contribute to cancer progression and metastasis (Xia et al., 2011; Ward et al., 2012), although their roles in the brain are poorly understood. The carboxyl terminal of ErbB2 carries a PDZ-binding motif and associates with a leucine-rich molecule, Erbin (Huang et al., 2001). As Erbin attenuates the activation of Ras-MAPK signaling linked to cell proliferation, its interaction with ErbB2 is implicated in oligodendrocyte differentiation and myelination (Tao et al., 2009; Dan et al., 2010; Liang et al., 2012).

Proliferating neural stem cells or precursors express high levels of ErbB2 (Abe et al., 2009a) in addition to ErbB1. Oligodendrocyte precursors express ErbB2 together with ErbB3, and ErbB2 activation contributes to the proliferation and differentiation of these cells via ErbB3 phosphorylation (Flores et al., 2000). A recent study revealed that signals from ErbB2:ErbB3 complexes in the neocortex regulate the expression of disrupted schizophrenia 1 (DISC1), which has been implicated in the genetics of schizophrenia (Seshadri et al., 2010). In hippocampal neurons, ErbB2:ErbB4 heterodimers influence the morphological differentiation of these cells (Gerecke et al., 2004).

## ErbB3 (HER3)

ErbB3 displays ligand preference for some members of the NRG family; ErbB3 has a high affinity interaction with NRG1, NRG2, and NRG6. Indeed, the intracellular domain of ErbB3 harbors more tyrosine residues that accept various adaptor/effecter molecules (Table 1). As its kinase activity is impaired, heterodimer formation with ErbB2 or the other ErbBs is essential to evoke signal transduction cascades. Alternative splicing of the *erbB3* gene produces soluble isoforms of ErbB3 (sErbB3) as well as isoforms with truncations in the intracellular domain (Lee and Maihle, 1998). Among these isoforms, p45 and p85 sErbB3s bind to NRG and decrease the effective concentrations of NRG1 in the extracellular space (Lee et al., 2001; Lin et al., 2008). The truncated isoforms of ErbB3 are found in cortical astrocytes and might be involved in attenuating NRG signaling (Citri and Yarden, 2006; Sharif and Prevot, 2010).

In the brain, high ErbB3 expression is only found in oligodendrocytes and their precursors. ErbB3 activation is involved in their propagation and differentiation (Makinodan et al., 2012). ErbB3 expression is also observed in neural precursor cells in the adult hippocampus and contributes to their proliferation, although ErbB3 expression is modest (Mahar et al., 2011). In human astrocytes, ErbB3, and ErbB1 form heterodimers that transduce NRG-dependent signals (Sharif et al., 2009). Again, EGF evokes NRG-like signaling through the dimerization of ErbB3 and ErbB1.

## ErbB4 (HER4)

**Figure 4:**
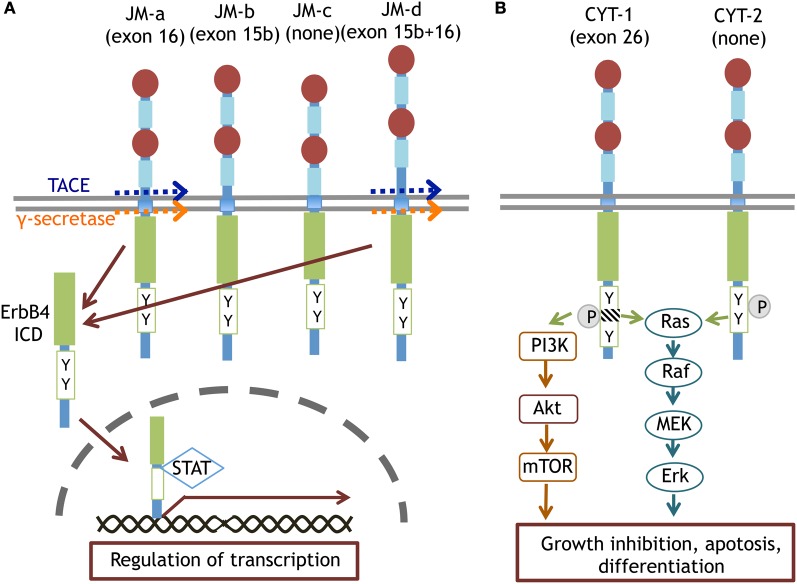
**Splice variants of ErbB4 proteins and their specific signaling.** The JM-a and JM-d isoforms of ErbB4 are cleaved by TACE or γ-secretase and converted to ErbB4-ICD. ErbB4-ICD is translocated into the nucleus with STAT and regulates gene transcription **(A)**. The CYT-1 and CYT-2 isoforms of ErbB4 differentially trigger Ras-MAPK signaling and/or PI3K-Akt signaling **(B)**.

ErbB4 mainly links to the Ras-MAPK and PI3K-Akt pathways. In contrast to ErbB1 signaling, ErbB4 phosphorylation induces sustained activation of the Ras-MAPK pathway, leading to cell cycle cessation and differentiation (Muraoka-Cook et al., 2008; Ortega et al., 2012). In the *erbB4* genome, alternative splicing of exon 15/16 and exon 26 produces ErbB4 variants, JM-a/b/c/d and CYT-1/2, respectively (Figure 4; Zeng et al., 2009; Veikkolainen et al., 2011). The phosphorylation of CYT-1 can recruit the p85 adaptor to activate PI3K-Akt signaling (Kainulainen et al., 2000). The CYT-1 sequence is susceptible to proteolytic cleavage by TNF-alpha converting enzyme (TACE) and γ-secretase (Vidal et al., 2005; Sundvall et al., 2010). Thus, ErbB4 proteolysis produces an 80 kD intracellular fragment (ErbB4-ICD) and liberates it into the cytoplasmic space. ErbB4-ICD interacts with the transcription factor STAT5 and migrates into the nucleus as a molecular chaperone (Vidal et al., 2005; Sundvall et al., 2010).

**Figure 5:**
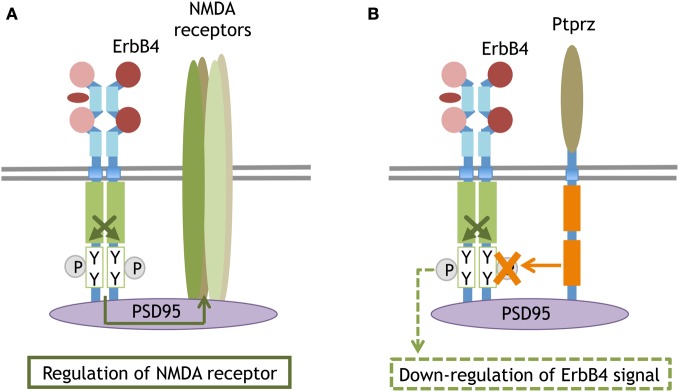
**Synaptic compartment of ErbB4 that binds to PSD95 or interacts with the phosphatase.** The scaffolding protein PSD95 anchors ErbB4 and the NMDA receptor at postsynaptic sites. The molecular interaction between ErbB4 influences NMDA receptor activity and function **(A)**. The PSD95-mediated interaction between ErbB4 and a membrane attached phosphatase, Ptprz. Ptprz eliminates the phosphate from ErbB4 and attenuates its signaling **(B)**.

ErbB4 also contains a PDZ-binding motif at the carboxyl terminal and is anchored to postsynaptic density protein 95 (PSD95) in neurons (Huang et al., 2000). Even when proteolytic cleavage produces ErbB4-ICD or when ErbB4 is phosphorylated with the ErbB partner, the signal is only minimally transported to the soma or translocated into the nucleus. Rather, the interaction with the scaffold protein PSD95 allows NMDA receptors to interact with ErbB4 and restrict ErbB4 signaling to the postsynaptic compartments (Garcia et al., 2000). Accordingly, impaired NRG1/ErbB4 signal is thought to underlie NMDA receptor dysfunction found in brain diseases such as schizophrenia (Hahn et al., 2006; Pitcher et al., 2011). ErbB4 also can form molecular complexes with the receptor-type tyrosine phosphatase (Ptprz) via its interaction with PSD95 (Fujikawa et al., 2007). In this complex, Ptprz interacts with ErbB4 as its substrate and de-phosphorylates ErbB4. Given the ligands for Ptprz (i.e., midkines and pleiotropins), NRG/ErbB4 signals can be disrupted by other cytokines through this receptor-type tyrosine phosphatase (Figure 5).

ErbB4 expression gradually increases in the brain and becomes pronounced in postmitotic neurons such as GABAergic interneurons, dopamine neurons, and cerebellar granule cells (Table 2; Elenius et al., 1997; Abe et al., 2009a; Vullhorst et al., 2009). The developmental pattern of ErbB4 expression is the opposite of that of ErbB1 (Abe et al., 2009a). ErbB4 signals may accelerate neural differentiation in these cell populations, potentially attenuating ErbB1 signaling (Woo et al., 2007; Fazzari et al., 2010). The NRG-driven up-regulation of glutamate receptor functions may represent the typical phenotypic responses of this cell population; ErbB4 activation regulates the activity and/or expression of both AMPA-type and NMDA-type glutamate receptors in GABAergic neurons (Gajendran et al., 2009; Abe et al., 2011; Ting et al., 2011). ErbB4 in midbrain dopaminergic neurons regulates the enzyme activity and expression of tyrosine hydroxylase *in vivo* as well as dopamine synthesis and release (Kato et al., 2011). Although ErbB4 is suggested to contribute to the migration and differentiation of immature GABAergic interneurons, these processes also involve ErbB1, and thus, the interplay between ErbB4 and ErbB1 needs to be characterized to reveal the full mechanism (Mahar et al., 2011; Li et al., 2012). In our previous studies, these phenotypic actions of NRG1/ErbB4 signals appear to be more modest than those of EGF/ErbB1 signals in neural cultures. Consistent with these findings, the gross brain structures and function of ErbB4-null knockout mice appear to be modest compared with ErbB1-null knockout mice (Sibilia and Wagner, 1995; Sibilia et al., 1998; Thuret et al., 2004). In this context, the crucial functions and/or biological significance of ErbB4 in the brain might not be fully characterized.

## Implications of abnormal ErbB signaling in brain diseases

ErbB signaling contributes to the development and maintenance of various cell populations in the central nervous system and is therefore implicated in the etiology or neuropathology of various brain diseases such as schizophrenia and Parkinson's disease, which involve cell dysfunction of GABAergic and/or dopaminergic neurons. Here we would like to discuss the potential pathological links between ErbB signaling and these brain diseases.

## Parkinson's disease

Parkinson's disease is a progressive neurodegenerative disorder in which patients exhibit obvious symptoms of motor dysfunction, such as shaking and muscle rigidity. This disease progresses to neurodegeneration of the midbrain dopaminergic neurons. Consistent with the neurotrophic actions of EGF on this cell population, the protein levels of EGF and ErbB1 are diminished in the postmortem brains of patients with this disease (Iwakura et al., 2005). A neurotrophic disturbance in ErbB1 signaling is reproduced in animal models of the disease; rats receiving a dopaminergic neurotoxin exhibit decreased ErbB1 and dopaminergic cell loss but EGF ameliorates these deficits (Pezzoli et al., 1991; Ventrella, 1993; Iwakura et al., 2005). Similarly, the contribution of ErbB4 signals to this illness is under investigation. Because of the higher blood-brain permiability of type 1 NRG1 (Kato et al., 2011), NRG1 was peripherally administeration to a Parkison's disease model to induce the neuroprotection of dopamine neurons (Zhang et al., 2004; Carlsson et al., 2011; Depboylu et al., 2012).

The molecular neuropathology of Parkinson's disease involves not only ErbB1 but also ErbB-interacting molecules. For example, LINGO-1, which associates with the Nogo-receptors in the nervous system, directly binds to ErbB1 to attenuate cell survival signals (i.e., PI3K-Akt signaling) in dopamine neurons (Inoue et al., 2007). Consistent with this finding, LINGO-1 expression is elevated in the substantia nigra of patients with Parkinson's disease. The molecule parkin, which is the causative gene for inheritable Parkinson's disease (Kitada et al., 1998), maintains ErbB1 signaling under normal conditions. Parkin can promote the ubiquitination and dissociation of Eps15 from ErbB1 to attenuate the internalization and degradation of ErbB1 (Fallon et al., 2006). Conversely, mutations in the parkin gene result in accelerated ErbB1 degradation, leading to the loss of neurotrophic ErbB1 signals in this disease.

## Schizophrenia

ErbB1 as well as ErbB4 is distributed in all the cell populations that are implicated in schizophrenia neuropathology, including GABAergic neurons, dopaminergic neurons, and glial cells. Several studies have focused on the ErbB1 molecule. Postmortem studies revealed that the ErbB1 protein is up-regulated in the forebrain regions of schizophrenia patients (Futamura et al., 2002). Animal studies demonstrate that acute and subchronic brain activation of ErbB1 triggers dopamine release in the striatum or globus pallidus, leading to behavioral impairments relevant to schizophrenia (Futamura et al., 2003; Tohmi et al., 2005; Mizuno et al., 2008; Sotoyama et al., 2011). In contrast to the effects on dopamine neurons, ErbB1 ligands negatively regulate GABAergic development in the neocortex and attenuate the activity of glutamate receptor channels in these neurons (Namba et al., 2006; Nagano et al., 2007). Conversely, quianozoline ErbB1 inhibitors can ameliorate schizophrenia-related behaviors in various animal models for schizophrenia (Mizuno et al., 2008). Both types of model studies indicate a pathological link between ErbB1 hypersignaling and schizophrenia. Given that ErbB1 and ErbB4 colocalization within the same neurons, it is likely that ErbB1 competes with NRG/ErbB4 signals, as was suggested in cancer studies (Moghal and Sternberg, 1999; Pitfield et al., 2006; Das et al., 2010).

Genetic studies have also demonstrated that schizophrenia is associated not only with the ligand NRG but also with its receptor ErbB4 (Stefansson et al., 2002). In 2006, SNP analysis revealed a genetic association between the *erbB4* gene and a particular type of splicing pattern associated with this illness (Norton et al., 2006; Silberberg et al., 2006). The risk of *erbB4* SNPs appears to correlate with the disease-specific spicing pattern (i.e., JM-a and CYT-1) in the prefrontal cortex and hippocampus of patients (Law et al., 2007; Tan et al., 2010). A postmortem study also found an increase in phosphorylated ErbB4 protein and its ability to form complexes with PSD95 but failed to detect a difference in total ErbB4 levels in schizophrenia patients (Hahn et al., 2006).

In addition to the neuropathology of GABAergic and dopaminergic neurons in schizophrenia, postmortem studies indicate the deficits in white matter and myelin structures are associated with this illness (Davis et al., 2003; Flynn et al., 2003). ErbB3 signals play crucial roles in oligodendrocyte myelination and saltatory conduction of nerve impulses (Stewart and Davis, 2004). Thus, several schizophrenia studies have focused on ErbB3 function. Aston et al. (2004) found that mRNA levels of genes related to myelin and oligodendrocytes, including *erbB3* mRNA, are down-regulated in the middle temporal gyrus of schizophrenia patients (Aston et al., 2004). However, the genetic association between *erbB3* SNPs and schizophrenia remains controversial (Kanazawa et al., 2007; Watanabe et al., 2007). In addition to the genetic association between *erbB* SNPs and schizophrenia, viral infection also directly triggers ErbB signaling and potentially contributes to brain mal-development.

## Impact of virokines on ErbB signaling

Virokine is a general term for a cytokine produced by viruses. By producing virokines, many viruses perturb the immune defense system of host organisms to escape clearance or promote host cell proliferation to enhance viral propagation (Klouche et al., 2004). A variety of virokines have been identified, including those that act on ErbB receptors (Table 3). In fact, some virokines are suggested to impair brain development (Billings et al., 2004). Thus, virokine production following viral infection directly influences brain development and might support the schizophrenia hypothesis of viral infection (Waddington and Buckley, 1996; Brown and Derkits, 2010).

**Table 3:** **Virokines that bind to ErbB receptors**.

**Virokine (ErbB-binding proteins)**		**Receptors**	**Related proteins**
VGF	VGF (Vaccinia virus growth factor, vaccinia 19kd protein)	ErbB1 homodimer	EGF, TGFα
	CGF (Cowpox growth factor)	ErbB1	VGF EGF family
	SPGF (Smallpox growth factor)	ErbB1	VGF EGF family
	SFGF (Shope fibroma virus growth factor)	ErbB1 containing dimer	VGF EGF family
	MGF (Myxoma virus growth factor)	ErbB2/ErbB3	VGF EGF family
E5 protein	Human papilloma virus type16 E5 protein	ErbB1 ErbB4 (JM-b CYT-1)	
	Human papillomavirus type6 E5 protein	ErbB1, ErbB2	
	Bovine papillomavirus type1 E5 protein	ErbB1	

**Figure 6:**
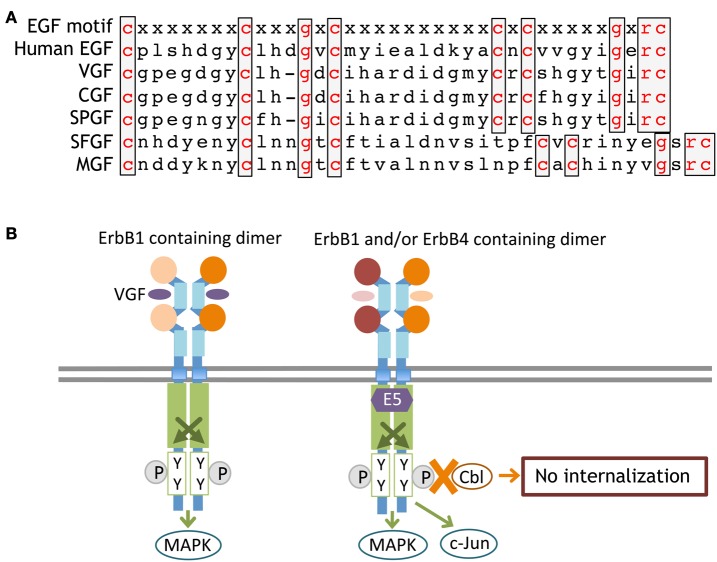
**Primary sequences of ErbB-interacting virokines and receptor interactions.** The primary amino acid sequences of EGF and virokines in the poxvirus family are shown **(A)**. An ErbB adaptor, E5, which is produced by papillomaviruses, associates with the kinase domain of ErbB and inhibits its internalization and/or degradation **(B)**.

Vaccinia virus growth factor (VGF, Vaccinia virus 19-kilodalton protein) is encoded by the genome of vaccinia virus in the poxvirus family and has an amino acid sequence homologous to EGF (Figure 6). VGF is produced and secreted from its membrane-anchored precursor and binds to ErbB1 receptors. VGF activates the Ras-MAPK pathway of host cells and promotes cell proliferation (Eppstein et al., 1985; Twardzik et al., 1985). Cowpox virus growth factor (CGF) displays high homology to VGF and enhances host cell propagation (da Fonseca et al., 1999). Additional EGF-like virokines have been identified in other pox viruses, including smallpox virus growth factor (SPGF; Kim et al., 2004), myxoma virus growth factor (MGF; Opgenorth et al., 1993), and shope fibroma virus growth factor (SFGF; Chang et al., 1987; Ye et al., 1988). These virokines also carry an EGF-like sequence and interact with ErbB receptors. According to the schizophrenia hypothesis of maternal and perinatal viral infection, the infection of these viruses and their translocation to the brain may perturb the normal development of dopaminergic or GABAergic neurons, although this assumption is fully hypothetical.

In addition to soluble virokines, virus-derived effectors can affect intracellular ErbB signaling. E5 is one of the early gene products of human papillomaviruses. E5 protein is also a membrane-anchored molecule, like the EGF precursor, but lacks the EGF-like domain. For example, the E5 protein of human papillomavirus type 16(HPV-16) associates with the ATPase motif of the ErbB1 tyrosine kinase domain, attenuate its interaction with Cbl, and inhibits the internalization and degradation of ErbB1 (Figure 6). Accordingly, papillomavirus infection enhances EGF/ErbB1 signaling to promote host cell proliferation (Chang et al., 2001; Venuti et al., 2011; Ganguly, 2012). In addition to ErbB1, the E5 protein also binds to the JM-b/CYT-1 isoform of ErbB4 and promotes host cell survival (Chen et al., 2007). The E5 protein in other papillomaviruses (human papillomavirus type 6 and bovine papillomavirus type1) exerts similar modifications on ErbB signaling (Martin et al., 1989; Cohen et al., 1993; Conrad et al., 1994). It is noteworthy that the human uterus can be infected with human papillomavirus type 16. Assuming that a human embryo develops in a uterus harboring papillomavirus, the potential direct or indirect impact of the E5 protein on fetal brain development cannot be overlooked.

## Provisional conclusion

Studies in cancer biology clearly indicate the pathologic powers of abnormal ErbB signaling and its contribution to oncogenesis, asthma, injury repair, and rheumatoid arthritis (Stoll and Elder, 1998; Davies et al., 1999; Satoh et al., 2001; Bersell et al., 2009; Calvo et al., 2010; Finigan et al., 2011; Yarden and Pines, 2012). In contrast to our knowledge of ErbB signaling in the periphery, the biological functions and regulation of ErbB signaling in the brain are still limited (Buonanno and Fischbach, 2001; Wong and Guillaud, 2004; Mei and Xiong, 2008). The ligand-bound ErbB receptor does not transmit signals, and instead the ErbB partner acts as a kinase substrate to trigger intracellular signaling. In addition, we need to consider which individual ErbB splicing isoforms are expressed in individual neural cells because some ErbB isoforms have a dominant-active or -negative function. Currently, we only know that *erbB* gene products are present in certain neurons or glia and not the real structures of particular ErbB isoforms in various brain regions. In this context, a more elaborate analysis may be required to accurately discuss their functions in the nervous system.

Although there are a total of four ErbB molecules, their ligands have significantly more diversity. There are six endogenous ErbB ligands in the EGF family and six in the NRG family. Virus-derived ErbB ligands also need to be considered. In contrast to the investigations on EGF or NRG1, the contribution of the other ligands, such as HB-EGF and NRG6 (neuroglycan C), is poorly understood even though those are highly expressed in the brain (Kinugasa et al., 2004; Nakanishi et al., 2006; Oyagi et al., 2009, 2011). Together with the proteolytic regulation of ErbB proteins and ligand precursors, ErbB signaling is regulated at multiple levels, including SNPs, alternative splicing, proteolytic processing, intracellular translocation, and signal cross talks between ErbBs. We hope this review will hint at the biological importance of ErbB in the nervous system and drive readers to challenge biological or pathological questions regarding ErbB signaling.

### Conflict of interest statement

The authors declare that the research was conducted in the absence of any commercial or financial relationships that could be construed as a potential conflict of interest.
